# Risperidone-Induced Neutropenia in a Schizophrenic Patient: A Case Report and Literature Review

**DOI:** 10.1155/2021/3980872

**Published:** 2021-08-17

**Authors:** Ayodele Atolagbe, Stanley Nkemjika, Olusegun Popoola, Oluwatoyin Oladeji, Irina Kogan, Haroon Saeed, Tolulope Olupona

**Affiliations:** ^1^Department of Psychiatry, Interfaith Medical Center, Brooklyn, NYC, NY, USA; ^2^Department of Population Health Sciences, School of Public Health, Georgia State University, Atlanta, GA, USA

## Abstract

Neutropenia is an adverse effect of various pharmacological therapies, including antipsychotics. Among the second-generation antipsychotic (SGA) medications, clozapine is most notable for neutropenic adverse effect. Risperidone, another SGA drug, is linked mainly with metabolic adverse effects, but rarely, blood dyscrasia adverse reactions have been reported. Hence, we report the case of a 56-year-old African American woman who developed severe neutropenia following two weeks of oral risperidone treatment. Her neutrophil levels returned to normal limits following discontinuation of risperidone and switching to haloperidol.

## 1. Introduction

Antipsychotic-related hematologic abnormalities have been reported in the literature [1] but remain a rare complication of some second-generation antipsychotic (SGA) medications [2]. This complication may be life-threatening as it exposes patients to infectious susceptibility following bone marrow suppression. Neutropenia, defined as a consistently low (<1500/*μ*L) absolute neutrophil count (ANC), can be due to reduced production or increased peripheral destruction [3]. The United States Food and Drug Administration (FDA) class warning on second generation antipsychotics reports possible adverse effect of blood dyscrasias, in the form of leukopenia, neutropenia, and agranulocytosis [4]. Hence, tricyclic antipsychotics such as the phenothiazines have been reported to cause neutropenia [5]. More notorious among the second-generation antipsychotic agents is clozapine, a dibenzodiazepine with a documented risk of agranulocytosis [6]. Though several antipsychotics might be related to leukopenia and neutropenia [7] risperidone, a benzisoxazole, has been reported to cause neutropenia in rare conditions [8]. It is noteworthy that the specific pathophysiology of these antipsychotic-induced neutropenias is still unclear [4, 9]. However, probable causes have been reported as stemming from the drug's effect on white blood cell (WBC) precursors, and as a result, protocols have been mandatory for regular monitoring of WBCs and ANCs included in computer-based registries of patients on clozapine treatment [6]. Though risperidone, olanzapine, and other antipsychotics do not have the same monitoring regulatory process as clozapine, neutropenia has been reported as a rare side effect of risperidone pharmacotherapy. Additionally, there is no evidence in the literature of risperidone-induced neutropenia in patients with past risperidone therapy exposure. Hence, we report a case of leukopenia and neutropenia occurring during treatment with risperidone in a chronic schizophrenic patient with comorbid hypertension and mild liver cirrhosis, which were resolved few days after the change of medication to a first-generation antipsychotic.

## 2. Case Report

A 48-year-old African American female was brought in by the emergency medical service on account of worsening agitation and aggressive behavior. The patient, who had a past history of hypertension and mild liver cirrhosis and a psychiatric history of schizophrenia, had been poorly compliant with her antipsychotic medications for the last two years. On presentation, the patient was very talkative; refused to follow safety instructions; exhibited poor frustration tolerance, poor impulse control, and poor reality testing; and appeared internally preoccupied. She endorsed auditory hallucinations and exhibited disorganized speech and behavior. A review of prior medication trial included mainly the first-generation antipsychotic (FGA) agents and risperidone therapy as the only second generation antipsychotic (SGA). She was subsequently admitted to the inpatient psychiatric unit and started on risperidone, with the plan of transitioning to a long acting injectable (LAI) paliperidone palmitate.

However, the patient refused to take any medications for the first 14 days of admission and was given emergency intramuscular injection of haloperidol 5 mg and lorazepam 2 mg pro re nata (PRN) several times following aggressive behavior in the unit. By the 14^th^ day of admission, the patient was commenced on treatment over objection court ruling management plan. The patient started taking risperidone orally disintegrating tablets 1 mg twice daily, which was gradually uptitrated to 3 mg twice daily by day 22 after admission. Following part of care management, patient's WBC count at presentation, on day 1, was 4,300/*μ*L [reference interval: 4500-11,000/*μ*L], ANC was 1800/*μ*L [Ref: 2000-7900/*μ*L], and platelet count was 258,000/*μ*L [Ref: 130,000-400,000/*μ*L]. On day 24, she was noted to have isolated neutropenia: WBC 2800/*μ*L, ANC 514/*μ*L. A repeated ANC on day 26 could not be estimated. During this time, the patient was only on risperidone. The patient was asymptomatic as she had normal vital signs, and there were no signs of extrapyramidal side effects. The patient's rapid plasma reagin (RPR) titer was within normal limit, SARS-COVID-19 polymerase chain reaction (PCR) test was not reactive, and hepatitis A and hepatitis B antibody titers were within normal limits. Her chest radio imaging and electrocardiogram reports were unremarkable. The patient's prothrombin time and international normalized ratio (INR) was 12.1 s [Ref: 9.8-13.4 s] and 1.04 [Ref: 0.85-1.15], respectively. Hemoglobin A1c (HbA1c) was 5.7% [Ref: 4.8-5.6%]. Her serum beta human chorionic gonadotropin (beta-hCG), serum electrolytes, blood urea nitrogen, creatinine, liver transaminases, and alkaline phosphatase were unremarkable. The patient's retroviral test was negative as well.

Risperidone therapy was discontinued on day 24 of her inpatient stay. The patient was commenced on FGA haloperidol 2 mg by mouth twice daily following termination of risperidone. The haloperidol dose was titrated to 5 mg twice daily and subsequently 10 mg twice daily due to persistent psychotic symptoms. Following the discontinuation of risperidone, monitoring of ANC on day 39 showed a progressive increase to 1100/*μ*L.

## 3. Discussion

Risperidone is a commonly used SGA in both the inpatient and outpatient psychiatric settings. Though there is documented evidence of adverse metabolic effects with risperidone pharmacotherapy, this was not evident in our patient. Instead, a neutropenia was evident, which resolved following termination of risperidone pharmacotherapy. Our patient did not have symptoms of acute viral illness and laboratory testing for other possible causes of neutropenia was negative. Cases of drug-induced neutropenia purely attributable to risperidone therapy are uncommon in the literature.

We conducted a review of the literature on the EMBASE, PSYCHINFO, and PubMed databases regarding the evidence on the adverse presentation of risperidone-induced leukopenia. The search results were suggestive that there is a gap in the literature on the topic as there are various reports on the relationship. Additionally, there are also synergistic and augmented pharmacologic effects on neutropenia. Evidence from literature shows that psychotropic drug-related neutropenia could be attributable to hereditary factors, commonly seen in the African American population and typically identified at an early age [10]. Other documented attributable factors are higher doses of neuroleptics, male gender, and neuroleptic naivety. Our case did not have the background attributable factors except for her racial origin. Based on these facts, this patient has been on risperidone treatment for multiple years without prior evident neutropenia; this suggests that the idea of race may not have been a probable cause of risperidone-induced neutropenia (RIN). Also, considering the chronicity of the patient's psychiatric condition with regard to her multiple hospitalizations, the concept of neuroleptic naivety does not apply to this case. Hence, an incidental finding of neutropenia a few days of commencement of risperidone therapy in the absence of any identifiable cause is exceptional.

In terms of pathophysiology, the documented body of evidence suggests that clozapine-induced neutropenia typically occurs within the first 18 weeks of treatment [11, 12], while RIN pathogenesis remains unknown and still needs further studies. Etiologically, using animal studies, it has been hypothesized that risperidone metabolites cause bone marrow suppression. Other postulated etiologies include decreased marrow neutrophil production, increased peripheral destruction of neutrophils by risperidone, and the role of the treated dendritic cell, which produced tumor necrosis factor-alpha- (TNF-*α*-) induced neutrophil death in vitro. Alternatively, this could also be attributable to immunomodulatory activities in genetically susceptible individuals, though this has not been fully explored in literature [13].

According to [14], patients with drug-induced neutropenia can typically continue such treatment if neutropenia is mild (above 1000/*μ*L) [14]. This guideline also applies to antipsychotic-related causes. Alternatively, medication dose tapering might be considered in benign neutropenia occurrence [14]. In this case report, considering the moderate to severe neutropenic state and the patient's comorbid medical problems—hypertension and mild liver cirrhosis, we switched from risperidone to a first-generation antipsychotic with a successful resolution. Other authors have reported successful resolution following a switch to another second-generation antipsychotic such as olanzapine [3, 15, 16]. Recombinant human granulocyte-cerebrospinal fluid therapy has been reported, and work up for other possible etiologies may be considered if neutropenia fails to resolve with other treatment modalities such as cessation of the causing agent [3, 15].

## 4. Conclusion

Risperidone-induced neutropenia is very unusual, and its incidence rate is unknown [3, 15–17]. Presently, there are no evidence-based alternative antipsychotic recommendations for incidental RIN. For the case presented here, we achieved a resolution of risperidone-induced severe neutropenia by changing to a haloperidol. Hence, a switch back to FGA medication could be an alternate treatment plan for such a scenario. Alternatively, olanzapine might also be a safe alternative in patients, who develop neutropenia on clozapine or risperidone. Additionally, olanzapine is ideal for adverse severe extrapyramidal symptoms from the first-generation neuroleptics [3, 15, 16].

Currently, there is no ANC monitoring requirement guideline for patients on risperidone treatment. Hence, we infer that routine monitoring of patients' ANC level on risperidone treatment irrespective of their hematological baseline might be good practice for all psychiatrists. We do recommend the extension of this practice to both the inpatient and outpatient service settings. Finally, clinicians should ensure routine hematologic evaluation for individuals with past or present histories of dyscrasias as part of the risperidone therapeutic management.
